# Further behavioural parameters support reciprocity and milk theft as explanations for giraffe allonursing

**DOI:** 10.1038/s41598-021-86499-2

**Published:** 2021-03-29

**Authors:** Markéta Gloneková, Karolína Brandlová, Jan Pluháček

**Affiliations:** 1 Department of Animal Science and Food Processing in Tropics and Subtropics, Faculty of Tropical AgriSciences, Czech University of Life Sciences Prague, Kamýcká 129, 165 00 Praha Suchdol, Czech Republic; 2 Department of Biology, Faculty of Science, Jan Evangelista Purkyně University in Ústí nad Labem, 400 96 Ústí nad Labem, Czech Republic; 3 Department of Ethology, Institute of Animal Science, Přátelství 815, 104 00 Prague-Uhříněves, Czech Republic; 4 Ostrava Zoo, Michálkovická 2081/197, 710 00 Ostrava, Czech Republic; 5 Department of Biology and Ecology, Faculty of Science, University of Ostrava, Chittussiho 10, 710 00 Ostrava, Czech Republic

**Keywords:** Behavioural ecology, Animal behaviour

## Abstract

Suckling of a non-filial calf, or allosuckling, is an extreme case of allomaternal care in mammals. There have been many hypotheses postulated in an attempt to explain this behaviour; however, the supporting evidence differs, together with the conclusions drawn from the investigated variables. Previously, suckling bout rejection was analysed, and the milk theft and reciprocity hypotheses were both determined as the most appropriate explanation of allosuckling in giraffe. In this study, seven hypotheses were tested using different behavioural parameters, namely suckling bout frequency, suckling bout duration, and time spent suckling. It is well-documented that these parameters are associated with various aspects in ungulate biology; for example, suckling rejection typically reflects milk intake and parent–offspring conflict, whereas the suckling bout duration and frequency is associated with social behaviours (affiliation, bonding, social stress). In total, 22 nursing females and 47 suckling calves were observed, in four Czech zoos during a five-year period. The correlation of the observed parameters between the reciprocal female-calf dyads was found to be in line with the reciprocity hypothesis. In addition, non-filial calves tried to steal the milk from non-maternal females, supporting the milk theft hypothesis. Thus, the results support both the reciprocity and milk-theft hypotheses as the most plausible explanation of allosuckling in giraffe, and illustrates the importance of using suckling bout duration and frequency, and the time spent suckling, as behavioural parameters that may aid in explaining the extremity of maternal investment, such as allosuckling.

## Introduction

Suckling is an essential part of maternal care, as it contributes to the important evolutionary process of infant survival and growth^1^, and remains an essential part of mother-infant bonding and maternal care from a behavioural point of view^2,3^. For mothers of various species, lactation may require energy demands that are higher than any other period in the life of the female^4,5^. The transfer of milk is an elementary part of reproduction in all mammals, yet species differ considerably with respect to suckling strategies^6–8^. Allosuckling (allonursing), which is the suckling (nursing) of non-filial young^9^, undoubtedly belongs amongst the most interesting suckling strategies^10–12^. Many hypotheses were proposed to explain the occurrence of allosuckling^9,13^, and these hypotheses can co-occur (i.e. they are not mutually exclusive)^13–17^. However, the evidence supporting these hypotheses remains limited, and only a few studies tested more than three of them simultaneously^18,19^.

The rate of allosuckling can be assessed by various parameters. The rejection rate of (allo)suckling is primarily used to reflect the energy intake via milk during suckling^7,19,20^. Other behavioural parameters are frequently used for the expression of maternal care, and they reflect the offspring’s demand rather than the rate of milk transfer^21–23^. These parameters are: suckling bout duration, and/or suckling bout frequency^8,24,25^. In addition, it was reported that in extreme cases, like allosuckling, these parameters could reflect parent–offspring conflict, as the bout duration may be connected with a shortage of milk, as well as maternal care for filial calves^21,22,26,27^.

In total, seven hypotheses that may explain allosuckling from a behavioural perspective are favoured by researchers. The reciprocity hypothesis (reciprocal altruism)^27–29^, predicts that females will allonurse the particular infants reciprocally. This theory assumes cooperation amongst individuals within one herd^13^. The female may receive greater benefits from having her offspring nursed by other females, while the costs of nursing a non-filial calf from the same herd may be much lower. Therefore, females may reciprocate the nursing the offspring of other females, and ensure that the members of the group will continue to nurse her filial offspring^13,30^. Typically, some individuals allonurse more than other females^10,30–32^.

The kin selection hypothesis^33^ suggests that females prefer to allonurse close kin, and therefore serve to improve their inclusive fitness^34^. The kin selection hypothesis represents the most common explanation of allosuckling in ungulates^9,13^. The parenting hypothesis assumes that inexperienced females may improve their ability to raise their filial offspring by allonursing^9,13^. The milk evacuation hypothesis^13^ states that females with surplus milk, which their filial offspring do not consume, are more likely to allonurse. The hypothesis of social benefits appears in societies with a social hierarchy, where the submissive females should preferentially allonurse the offspring of dominant females^13,35^. The misdirected care hypothesis states that a female allonurses a non-filial offspring because she is not able to discriminate her own offspring from a non-filial one, and does not recognize that the suckling offspring is not her own^36–38^.

The milk theft hypothesis has been mostly connected with misdirected care hypothesis^13^, and some studies do not distinguish between these two situations^31^. However, the milk-theft hypothesis explains the behaviour of the calf, and not that of the nursing female^19^. The offspring tries to steal the milk from a nonmaternal female by suckling together with the filial young, in a specific position that is further from the female's head, and thus smell, to compensate for insufficient milk intake from its own mother, or to simply get milk^9^. Together with the kin selection hypothesis, the milk theft hypothesis remains one of the most common explanations of allosuckling in mammals^14,18,19,31,39,40^.

Allosuckling in giraffes has been recorded in the wild^2,41–43^, and the nursing bout duration was highly variable (4–360 s)^2^, as well as in captivity^2,10,19,21,44^. Based on the rejection rate, a high occurrence of allosuckling was documented in captive giraffe^19^. Evidence was found in support of the milk-theft and the reciprocity hypotheses as the two most influential causes, in this case^19^. Therefore, in the present study, other behavioural variables associated with allosuckling were focus on, namely suckling bout frequency, suckling bout duration, and time devoted to nursing (from the female’s perspective)/time devoted to suckling (from the calf's perspective). Seven hypotheses were tested—the kin selection hypothesis, the reciprocity hypothesis, the misdirected care hypothesis, the milk theft hypothesis, the hypothesis of social benefits, the parenting hypothesis, and the milk evacuation hypothesis (Table 1).

**Table 1:** Hypotheses and predictions of allosuckling in giraffe.

Hypothesis	Prediction	Statistical model
Kin selection	Allosuckling bout frequency in the female-calf dyad will increase with increasing kinship	Model 4
Reciprocity	Allosuckling bout frequency in the female-calf dyad will correlate with reciprocal dyads	Model 5
Misdirected care	The suckling bout duration of the filial calf will not be longer than those of non-filial ones The bouts will not last longer when an antiparallel position was adopted compared to two other positions Female sniffing the calf during the bout will not affect the suckling duration accordingly (shorter for non-filial and longer for filial)	Model 1
Milk theft	The suckling bout duration of the filial calf will be longer than that of non-filial ones More calves within one bout, and the bout will be shorter The bouts will last longer when antiparallel position will be adopted compared to two other positions The time devoted to allosuckling will not decrease with the increasing age of non-filial calves	Model 1 and Model 2
Social benefits	The allosuckling bout duration will be shorter when the mother’s rank is lower than that of the allosuckling female	Model 1
Parenting	Suckling bout duration and frequency will be shorter/lower in multiparous mothers than in primiparous ones	Model 1 and Model 3
Milk evacuation	Allosuckling bout frequency will increase with the increasing age of filial calves Unsuccessful allosuckling attempts will decrease with the increasing age of fillial calf	Model 1 and Model 6

## Results

### Suckling bout duration

The average suckling bout duration lasted 19.60 ± 14.18 s (*N* = 1139 bouts). The average suckling bout of filial calf persisted for 20.97 ± 15.85 s (*N* = 564 bouts), and the average allosuckling bout of non-filial calf lasted 18.26 ± 12.18 s (*N* = 575 bouts). The longest suckling bout of a filial calf was 2 min. and 57 s., and the longest allosuckling bout of a non-filial calf was 1 min. and 32 s.

#### 1a. Suckling bout duration terminated by mother

Only bouts terminated by the mother were included (*N* = 752 bouts) into the model. The bouts of the filial calf were longer than that of the allosuckling bouts of non-filial calves (*F*_*1,717*_ = 5.32; *P* = 0.021). The suckling bout duration was affected by the position of the suckling calf (*F*_*2,717*_ = 5.56; *P* = 0.004),with the bout lasting longer when the calf adopted an antiparallel position, compared to if they were in a parallel position (*t*_*717*_ = 3.22; *P* = 0.004) or in a position behind the female (*t*_*717*_ = 2.49; *P* = 0.034). The suckling bout duration was affected by sniffing of the female during the bout (*F*_*1,717*_ = 17.30; *P* < 0.001), and when the female sniffed the calf, the suckling bout lasted longer than when she did not sniff it. Furthermore, the suckling bout duration was affected by the number of suckling calves within the bout (*F*_*1,717*_ = 75.37; *P* < 0.001), with the duration of the suckling bout decreasing with the increasing number of calves suckling simultaneously within the bout. Finally, the herd identity (*F*_*12,717*_ = 3.41; *P* < 0.001) affected the suckling bout duration. No other factor was significant.

#### 1b Allosuckling bout duration

**Figure 1:**
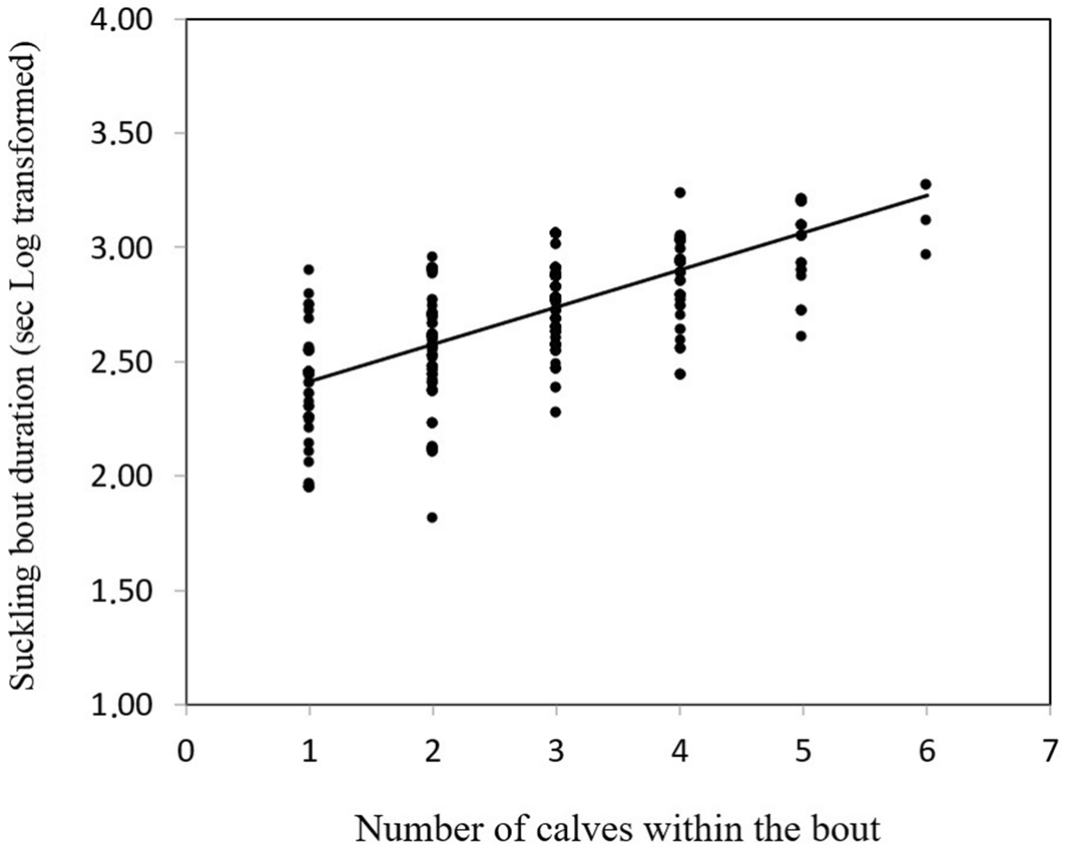
Allosuckling bout duration increased with the increasing number of calves in the bout.

When only allosuckling was included in the analysis (*N* = 575 bouts), the allosuckling bout duration was affected by the number of suckling calves (*F*_*1,545*_ = 46.73; *P* < 0.001), and the duration of the allosuckling bout increased with an increasing number of suckling calves (Fig. 1). Furthermore, the allosuckling bout duration was affected by the hierarchical rank difference between the nursing female and the calf’s own mother (*F*_*1,144*_ = 5.62; *P* = 0.019). The allosuckling bout duration was longer when the allosuckling female was ranked higher in the hierarchy than the mother of the suckling calf, than when she ranked lower than the mother of the calf.

### Time devoted to suckling

Calves devoted an average of 3.15 ± 5.07 s of suckling per hour of observation (*N* = 1279). The filial calves devoted on average 3.78 ± 5.87 s to suckling per hour of observation (*N* = 585), and the non-filial calves performed 2.62 ± 4.21 s of suckling per hour of observation (*N* = 694).

Time devoted to suckling was also affected by the relatedness (*F*_*1,1218*_ = 44.51; *P* < 0.001) and age (*F*_*1,1218*_ = 38.35; *P* < 0.001) of the calf. The total time devoted to suckling decreased with an increasing age of filial calves, but it was unaffected by increasing age in non-filial offspring (*F*_*1,1218*_ = 29.38; *P* < 0.001).

### Time devoted to nursing

The mean time devoted to nursing was 5.32 ± 6.97 s per hour of observation (*N* = 491). The females devoted 4.50 ± 6.22 s per hour (*N* = 491) to nursing filial calves, and 3.38 ± 5.04 s per hour to the allonursing of non-filial calves (*N* = 491).

**Figure 2:**
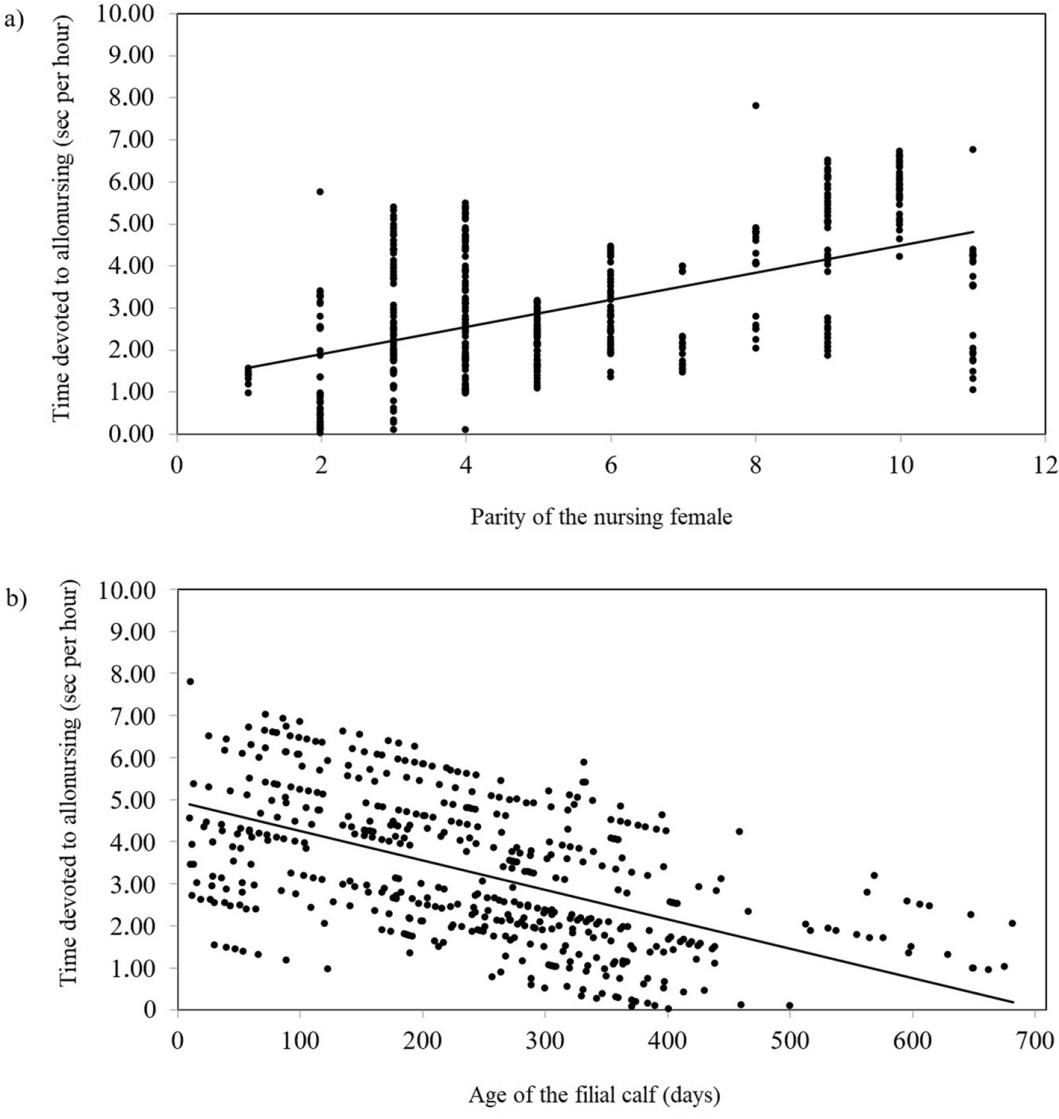
The time which a female devoted to allonursing increased with the increasing parity of the female (**a**), while it decreased with the increasing age of the filial calf (**b**).

The mean time devoted to the allonursing of non-filial calves was 3.22 ± 4.34 s per hour (*N* = 487). The time devoted to allonursing increased with the increasing parity of the nursing female (*F*_*1,460*_ = 11.19; *P* = 0.0009; Fig. 2a), but it decreased with the increasing age of the filial calf (F_1,460_ = 19.97; *P* < 0.001; *N* = 487; Fig. 2b), and was affected by the herd identity (*F*_*5,460*_ = 2.71; *P* = 0.0136).

### Female-calf dyads

#### Allosuckling bout frequency/time devoted to allosuckling

The average allosuckling bout frequency in female-calf dyads was 0.048 ± 0.070 (range 0–0.4; *N* = 153 dyads) events per hour of observation. Allosuckling bout frequency increased with increasing kinship (*F*_*1,124*_ = 9.90; *P* = 0.002), as well as with increasing parity of the female (*F*_*1,124*_ = 9.61; *P* = 0.002). Allosuckling bout frequency in female-calf dyads was affected by the herd identity (*F*_*6,124*_ = 3.77; *P* = 0.002). Similarly, the time spent allosuckling in individual dyads increased with increasing kinship (*F*_*1,124*_ = 5.87; *P* = 0.017), as well as with increasing parity of the female (*F*_*1,124*_ = 10.44; *P* = 0.002), and was affected by the herd identity (*F*_*6,124*_ = 4.75; *P* < 0.001).

#### Reciprocity

**Figure 3:**
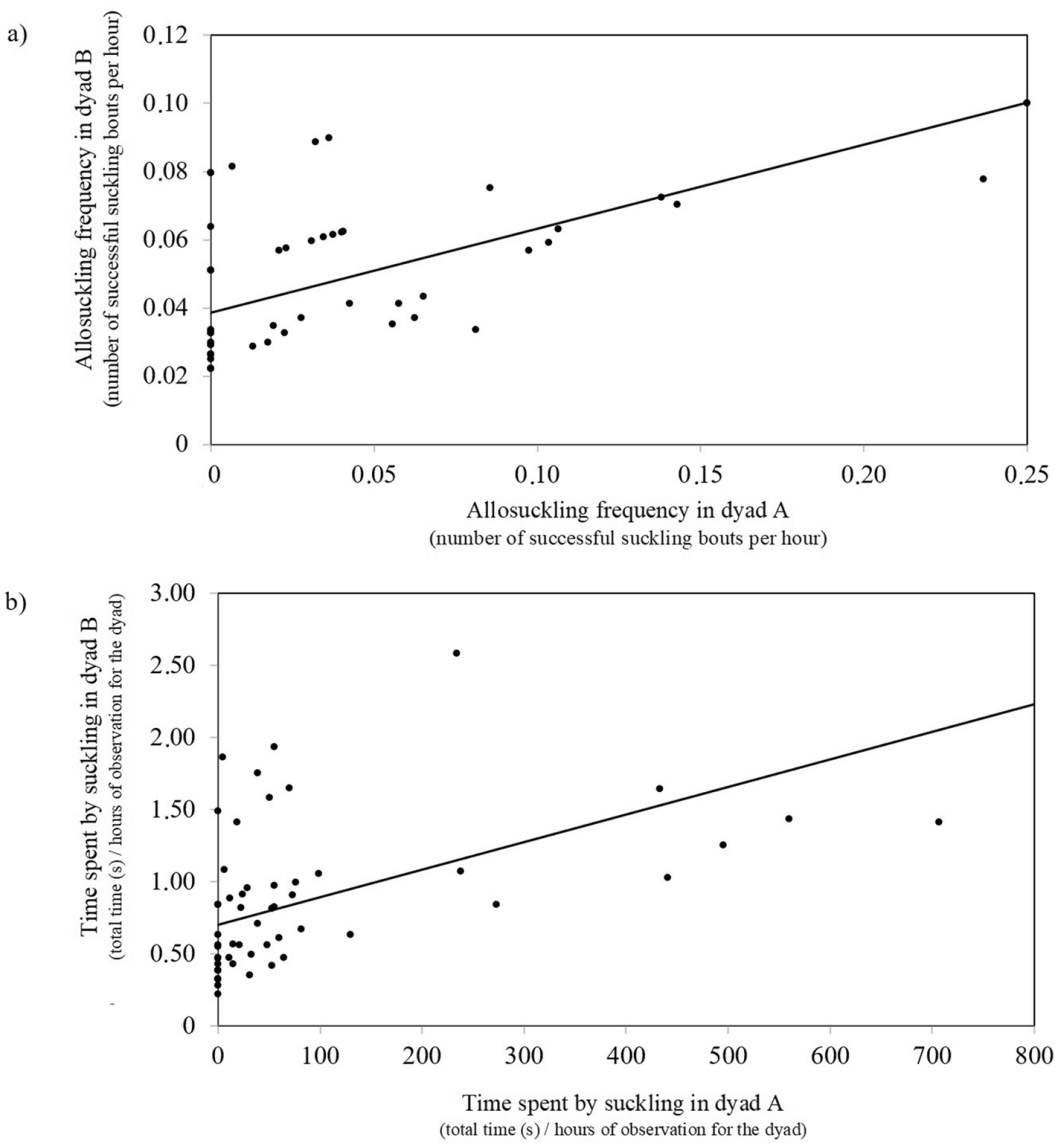
Allosuckling frequencies increased for both dyad A and B when compared (**a**). The same applied for the time devoted to allosuckling in the respective dyads (**b**).

Allosuckling frequency in reciprocal dyads (*N* = 58 dyads) were significant (*F*_*1,38*_ = 5.56; *P* = 0.024; Fig. 3a), such that when allosuckling frequency was higher in dyad A, it was also higher in dyad B. Similarly, the time devoted to allosuckling in dyad A increased concomitantly with that in dyad B (*F*_*1,38*_ = 12.45; *P* = 0.001; Fig. 3b). No other factor was significant in the models analysed. On the other hand, the frequency of allosuckling attempts in dyad A was not affected by the frequency of that in dyad B.

### Unsuccessful (allo)suckling attempts

The average frequency of unsuccessful suckling attempts per hour was 0.41 ± 0.55 (*N* = 1279). The average frequency of unsuccessful suckling attempts for filial calves was 0.55 ± 0.67 (*N* = 585), and 0.29 ± 0.40 per hour (*N* = 694) for non-filial calves. The highest frequency of unsuccessful bouts was 6 times per hour for filial calves, and 3.75 times per hour for non-filial calves.

Unsuccessful suckling attempt frequencies were affected by the degree of relatedness. The frequency of unsuccessful suckling attempts was higher for filial calves than for non-filial ones (*F*_*1,1191*_ = 97.91; *P* < 0.001), and was affected by the age of the calf (*F*_*1,1191*_ = 93.79; *P* < 0.001). The frequency decreased with an increasing age of the filial calf but did not change according to the age of non-filial calf (*F*_*1,1191*_ = 33.80; *P* < 0.001). The frequency of unsuccessful suckling attempts was affected by the identity of the herd (*F*_*5,1191*_ = 8.61; *P* < 0.001).

## Discussion

In this study, it was found that giraffe allonursed non-filial calves reciprocally, further supporting the reciprocity hypothesis as the primary explanation for allonursing amongst captive giraffe. While this hypothesis did not receive much support in the past^13^, recent studies involving ungulates provided evidence in support of reciprocity, explained by allosuckling (*Equus grevyi*^32^; *Rangifer tarandus*^28^; *Giraffa camelopardalis*^19^, *Bison bison*^29^). The current study revealed that allosuckling frequencies and time devoted to allosuckling increased concomitantly in dyad A and dyad B, corresponding with the reciprocity hypothesis which assumes that two females achieve higher fitness when nursing each other's offspring. Although, previously no correlations were found between the rates of allosuckling in reciprocal dyads^19^; however, the present study used different behavioural variables (suckling bout duration and suckling bout frequency). The reason for these contrasting results may be explained by the fact that while calves try to allosuckle from various females regardless of reciprocity, the final decision allowing them to allosuckle is made by the females, reflecting reciprocity.

Allosuckling bout frequency, as well as the time devoted to allosuckling, increased with increasing kinship in the present study; however, when both variables (kinship and reciprocity) were used in the model, the AIC criteria favoured reciprocity. Similarly, the study of allogrooming in primates tended to favour reciprocity over kinship^45^. Reciprocity and kinship are, however, very difficult to separate, especially in herds formed by relatives. The effect of kin selection was not supported in a previous study of giraffe either^19^. However, kin selection may correspond with the social structure of the giraffe herd, which represents a fission–fusion social system. This system occurs within a large community where the individuals create subgroups, changing in size and composition every day^46–49^. The social ties in the fission–fusion society may be influenced by kinship^48^ and thus it is necessary to continue to test kinship as a possible explanation of allonursing in giraffe.

The duration of suckling of the filial calf was longer than the duration of allosuckling in non-filial calves, but the nursing duration was longer when the calf approached from the head of the female and when the female sniffed the calf, both when the calf was filial or not. Therefore, it can be assumed that the females are able to identify, and prefer, the filial calf. In line with this result, it may be deduced that calves thus try to steal milk, but the female can identify them and decides whether to nurse them or not (or rather, they do not misdirect the care).

These results thus also support the milk theft hypothesis, which is one of the most supported hypotheses in captive giraffe^19,44^, as well as in other ungulates, including water buffalo^31^, camels^39^, guanacos^40^, red deer^15^, or reindeer^17^. It was initially hypothesized that the duration of allonursing would decrease with an increasing number of suckling calves, as the female would prefer to prevent the allosuckling of additional non-filial calves. However, the allosuckling duration increased with an increasing number of suckling calves in the present study, which implies that the longer the nursing bout continues, the more calves have the chance to realize the event and join it. In most cases, multiple nursing was initiated by the suckling of the filial calf, and once the female decided to nurse the filial calf, she chose not to stop nursing when the non-filial calves joined. The same result was found in camels^39^, where the nursing duration was longer for multiple nursing than when the female was nursing only one calf.

In contrast to the hypothesis of social benefits, the allosuckling bout duration was longer when the allonursing female ranked higher in the hierarchy than the mother of the suckling calf, than when she ranked lower, the tendency of which is already reported in giraffe^19^. The explanation for this result could be that the rank of the female is associated with their body condition, as a higher ranking female is more likely to be granted access to food^50,51^. The dominant female could therefore be in better condition, and the costs for allonursing are proportionately low, or the female may even produce a surplus of milk compared to the more submissive one.

Moreover, the time devoted to (allo)nursing increased with the increasing parity of the female, and thus the more experienced females allonursed more than the less experienced ones, which contradicts the parenting hypothesis. In previous studies, a high effect of the parity on the nursing behaviour of captive giraffe was found; more specifically, the probability of successful suckling, the acceptance rate within the female-calf dyad^19^, as well as the allonursing bout frequency, and the total time devoted to nursing in all events (filial and non-filial), increased with increasing female parity^10^. Females are expected to change the rate of maternal care with their reproductive experience^52,53^, although, it can be can assumed that more experienced females are able to cope better with allosuckling and milk distribution, and realise that providing additional milk to other calves has no deleterious effect on their current filial offspring.

Furthermore, no support for the milk evacuation hypothesis was found, as when the filial calf was younger, the time devoted to (allo)nursing was longer, in the present study. This result corresponds with the progression of the lactation phases, that the amount of milk at the initiation of lactation and nursing is higher and the female has enough resources/milk to (allo)nurse. However, the need, as well as the amount of maternal milk, decreases with the increasing age of the filial calf. The same result was found from the calf's point of view, when the time devoted to suckling, as well as the frequency of attempts, decreased with the age of the calf and with their need of maternal milk. On the other hand, time devoted to allonursing, time devoted to suckling, and the frequency of suckling attempts did not decrease with the increasing age of the non-filial calf, and thus the non-filial calves try to allosuckle (to steal) regardless of their age, which aligns with the milk theft hypothesis.

In the current study, suckling bout frequency, suckling bout duration, and time devoted to nursing/time devoted to suckling was used to test the hypotheses explaining allonursing. Many studies in mammals that correlated the suckling bout frequency and time devoted to suckling with milk intake, including feral horses (*Equus caballus*)^54,55^, fallow deer (*Dama dama*)^56^, domestic mice (*Mus domesticus*)^24^ and domestic cats (*Felis catus*)^24^, did not show a significant relationship between the suckling bout duration and milk or energy intake. Nevertheless, it was demonstrated that these variables could reflect the behavioural aspects of maternal care^22,26^, and it should be stressed that similar results were found both within the current study, and the previous giraffe study evaluating the suckling rate^19^. Thus, the current results demonstrate that suckling bout duration and frequency, in extreme cases such as allosuckling, could be associated with the conflict between the female and the offspring^21,26^.

Allonursing has been mostly observed in captivity^9^, which could be associated with a higher population density and unlimited access to food. On the other hand, allonursing in giraffes has also been observed in the wild^2,41–43^, where the conditions for the observations are far more difficult. Thus, in the case of giraffe, the captive conditions might amplify the pre-adaptation to allonurse. Furthermore, effect of captivity does not explain why the giraffe allonurse reciprocally.

This study supports previous results explaining the high extent of allonursing in giraffe. Using suckling bout frequency, suckling bout duration, and time devoted to nursing/time devoted to suckling, as behavioural measurements, the role of reciprocity in allonursing is extended to giraffe. While the non-filial calves are actively trying to steal milk, the females are able to identify them, and still, the female continues to allonurse, even showing increased allonursing with increasing experience of the female (parity). Furthermore, the kin selection hypothesis cannot be excluded, as giraffes are generally known to prefer closely related individuals as herd mates.

## Material and methods

### Ethical note

The ethological observation of the giraffe in zoos always took place from the area designated for visitors. As the animals were habituated to the presence of people in this area, the observer did not disturb them, or influence their common behaviour. The observations did not disrupt the daily management of the stables. The research was approved by the head zookeepers and managers responsible for the animals in every zoo where data collection occurred.

### Study animals

In total, 22 nursing females and 47 suckling calves of Rothschild’s giraffe (*G. c. rothschildi*), recently classified as Nubian giraffe (*Giraffa c. camelopardalis*)^57^, were observed in four zoological gardens in the Czech Republic: Praha Zoo (seven females and 26 calves, in 2007–2013), Safari Park Dvůr Králové (10 females and 13 calves, in 2008 and 2011), Olomouc Zoo (three females and four calves, in 2011), and Liberec Zoo (two females and four calves, in 2008). The observation started when two or more calves were born. The number of calves present in the herds ranged from two to eight. The giraffe were identified individually by their sex, coat pattern, body size, and shape of the horns and hooves^58^.

The giraffe in all four zoos were kept in stables during the winter, and they were released into outdoor enclosures during the day, when the weather allowed for this. All of the animals had ad libitum access to hay and branches, and limited supplementation with granulated food, fruit, and vegetables. Pregnant females (before, during and after parturition) were fed separately, with lactation supplements.

### Data collection

The data were collected by the ad libitum sampling of all suckling events in the herd^59^. The observations took place every 7–14 days. The research study was terminated when the calves were naturally weaned (approximately 12 months of age) or separated from the mothers according to the management of the zoo according to the decision of the breeding programme coordinator. Each observation session lasted for 3–6 h, based on the possibilities provided by the zoo management.

All suckling events were recorded, including successful suckling bouts and unsuccessful suckling attempts. An unsuccessful suckling attempt was defined as when the calf took the teat into their mouth for less than 5 s^10,19,21,23^, or when the calf just approached the udder of the female^10,19,60^. A successful suckling bout was defined as when the calf held the teat in the mouth for 5 s or longer^10,19,21^. The suckling bout was considered to be complete when the calf stopped suckling for more than 10 s^10,19,21,23^.

### Statistics

All of the data were analysed using SAS System, Version 9.4. Factors affecting the suckling bout duration, frequency, and the time devoted to suckling/time devoted to nursing were tested, using multivariate general linear mixed models (GLMM, PROC MIXED, SAS).

#### Model 1ab—suckling bout duration

In the first model the following independent variables (fixed factors) were analysed: age of the calf (in days), age of the calf’s own mother (5–23 years), female’s gravidity (yes, no), herd identity, the hierarchy rank difference between nursing female and calf’s own mother (lower, higher), the initiator of the bout (calf, female), the number of suckling calves within the suckling bout (1–6), parity of the nursing female (1–11), the place where the suckling bout occurred (stable, enclosure), the position of suckling calf (antiparallel/perpendicular, parallel, and from behind), the presence of the adult male in the herd (yes, no), relatedness (filial, non-filial calf), sex of the calf, the side of the female where the calf approached (left, right), if the female sniffed the calf during the suckling (yes, no), the animal who terminated the bout (calf, female, male), and their first-order interaction terms. The female’s and calf’s identities were used as the random factors.

The analysis of suckling bout duration was performed in two models. The first model (Model 1a) contained all abovementioned independent variables and limited the data set to bouts that were terminated by the female. The second model (Model 1b) focused only on allosuckling bouts. Log transformation of suckling bout durations to approach normal distribution were performed; in addition, all bouts longer than 100 s (n = 6) were excluded, to prevent the effect of outliers on the analyses.

#### Model 2, 3—the time devoted to suckling/time devoted to nursing

The total time devoted to suckling (from the calves point of view), or time devoted to nursing (from the females point of view), was defined as the time devoted to allonursing by each individual female per hour of observation, and the time devoted to allosuckling by each individual calf per hour of observation, respectively.

Analyses of the time devoted to suckling (Model 2) included the following fixed factors: relatedness, the age of the calf, the sex of the calf, calf order, the herd identity, and their first-order interaction terms. The calf identity was included as a random factor in these analyses. Times devoted to suckling longer than 25 s (n = 8) was excluded, in order to avoid the effect of outliers from the respective analyses.

In the analyses of the time devoted to nursing (Model 3), the following fixed factors (independent variables) were involved: relatedness (filial, non-filial calf), herd identity, parity of the nursing female (1–11), the age of the nursing female (5–23 years), hierarchical rank of the nursing female (1–6), and the female’s gravidity (yes, no). Again, the random factor in the model was the calf identity. Times devoted to nursing that were greater than 25 s (n = 15) were removed, to avoid the effect of outliers in this model.

#### Model 4, 5—female-calf dyads

To verify the kin selection and the reciprocity hypotheses, the allosuckling bout and attempts frequency, and the time devoted to suckling in female-calf dyads were analysed. For each possible dyad (dyad A; Model 4), the suckling bout frequency was counted as a frequency of successful suckling or allosuckling bouts for each individual dyad per one hour of observation. The total time devoted to nursing was defined as the time devoted to nursing/allonursing within each dyad (total time (s)/hours of observation).

‘Kinship’, which included Wright's coefficient of relatedness (r)^61^ calculated for each calf-female dyad based on studbook data, was used. Using a general linear model (GLMM, PROC MIXED, SAS), it was tested whether the suckling bout frequency, as a frequency of successful suckling or allosuckling bouts in the dyad, was affected by kinship, female parity and/or herd identity.

To test reciprocity (Model 5), the suckling bout frequency was included as a frequency of successful suckling or allosuckling bouts of the reciprocal dyad (dyad B; e.g. dyad A = calf A and female B, and the reciprocal dyad B = calf B and female A) among the independent variables. To avoid duplicates, the data set was limited to represent each dyad pair only once.

#### Model 6—the frequency of suckling attempts

The frequency of suckling attempts involved the number of unsuccessful attempts (= rejected by the female) performed per one hour of observation for each individual female, or by each individual calf.

Analyses of suckling attempts frequency (Model 6) included the following fixed factors: relatedness, the age of the calf, the frequency of suckling attempts (in the analysis of suckling bout frequency, only), the sex of the calf, the herd identity, calf order, and their first-order interaction terms. The calf identity was included as a random factor in these analyses. Frequencies of attempts higher than 2 (n = 32) were excluded, to avoid the effect of outliers from the analysis.

In all analyses, the full model was first investigated, including all of the fixed effects. The significance of each of the fixed effects in the mixed model were assessed via *F*-tests. The non-significant fixed effects were sequentially removed, unless they improved the quality of the model (using AIC criteria for comparing the models).

To avoid problem with pseudoreplications from the same individual, the individual female was nested within the herd (in the analyses of suckling bout duration) or the individual calf (in analyses of suckling bout frequency and time devoted to suckling) was included as a subject in the *repeated* statement (GLMM, SAS). The within-group means were appropriately adjusted for the other effects in the model (LSMEANs statement). For multiple comparisons between means, *t*-tests were applied using Tukey–Kramer adjustment.

## Data Availability

Data from this study can be made available upon request.
